# The Protein Kinase aPKC as Well as the Small GTPases RhoA and Cdc42 Regulates Neutrophil Chemotaxis Partly by Recruiting the ROCK Kinase to the Leading Edge

**DOI:** 10.1111/gtc.70002

**Published:** 2025-02-05

**Authors:** Atsushi Naito, Sachiko Kamakura, Junya Hayase, Akira Kohda, Hiroaki Niiro, Koichi Akashi, Hideki Sumimoto

**Affiliations:** ^1^ Department of Biochemistry Kyushu University Graduate School of Medical Sciences Fukuoka Japan; ^2^ Department of Medical Education Kyushu University Graduate School of Medical Sciences Fukuoka Japan; ^3^ Department of Medicine and Biosystemic Science Kyushu University Graduate School of Medical Sciences Fukuoka Japan

**Keywords:** aPKC, Cdc42, chemotaxis, neutrophil, RhoA, ROCK1

## Abstract

The small GTPases RhoA and Cdc42 and their effector proteins play crucial roles in neutrophil chemotaxis. However, endogenous localization and regulation of these proteins have remained largely unknown. Here, we show, using a trichloroacetic acid fixation method, that endogenous RhoA and Cdc42 are preferentially accumulated at the F‐actin‐rich leading edge (pseudopod) during chemotaxis of human neutrophil‐like PLB‐985 cells in response to the chemoattractant C5a. Interestingly, the enrichment of RhoA is impaired by knockdown of Cdc42, indicating a positive regulation by Cdc42. Depletion of Cdc42 or RhoA each induces the formation of multiple pseudopods, confirming their significance in cell polarization with an organized actin network at the front. The Rho‐associated kinase ROCK is also recruited to the leading edge during chemotaxis in a manner dependent on not only RhoA and Cdc42 but also aPKC, a Cdc42‐interacting kinase that can also bind to ROCK. ROCK promotes phosphorylation of the myosin light chain at the front, possibly regulating pseudopod contractility. Knockdown of aPKC suppresses neutrophil chemotaxis by disturbing pseudopod orientation without forming multiple protrusions. An incorrectly oriented pseudopod is also observed in ROCK‐depleted cells. Thus, aPKC, as well as RhoA and Cdc42, likely regulates neutrophil chemotaxis partly by recruiting ROCK to the leading edge for correct directionality.

## Graphical abstract

Endogenous RhoA is accumulated during chemotaxis to the leading edge of neutrophil‐like PLB‐985 cells, which are fixed with trichloroacetic acid before immunofluorescence imaging (left). Endogenous ROCK1 is also recruited to the leading edge in a manner dependent on RhoA and aPKC (right) as well as Cdc42. In conjunction with RhoA and Cdc42, the cell polarity protein aPKC likely regulates neutrophil chemotaxis partly by recruiting ROCK to the leading edge for correct directionality.

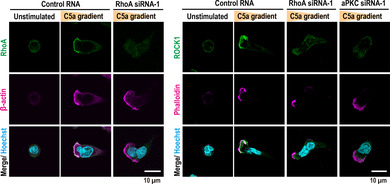

## 1 Introduction

Neutrophils are the predominant leukocytes in circulating human blood and serve as the first and crucial responder in host defense, rapidly migrating from the blood to infected or injured (inflamed) sites for killing pathogenic microbes and removing cellular debris (Burn et al. 2021; Nauseef 2023). The directed neutrophil migration to the target sites is conducted by a process known as chemotaxis, which is triggered by a variety of soluble chemoattractants, including the pathogen‐derived *N*‐formyl peptide fMLP and the complement‐derived anaphylatoxin C5a (Lawson and Ridley 2018; SenGupta, Parent, and Bear 2021).

Motile cells coordinate actin cytoskeleton dynamics at the front and back to achieve directed migration (Lawson and Ridley 2018; SenGupta, Parent, and Bear 2021). In response to diffusible chemoattractants, neutrophils undergo cell polarization via cytoskeletal rearrangement with an F‐actin‐rich leading edge (pseudopod) at the front and a myosin II‐rich trailing end (uropod) at the rear. In a prevailing model, the small GTPases Cdc42 and Rac are both activated at the front and stimulate actin polymerization via their respective effectors WASP (Kumar et al. 2012; Brunetti et al. 2022) and WAVE (Weiner et al. 2007; Millius et al. 2009), leading to pseudopod formation, whereas RhoA, another related small GTPase, is most active in the body and rear of migrating cells to regulate actomyosin contraction and rear retraction (Lawson and Ridley 2018; SenGupta, Parent, and Bear 2021). The motive force is produced by actin‐mediated front protrusion and myosin IIA‐driven rear contraction, although it is thought that neutrophils rely largely on their protrusive fronts to drive cell migration (Hadjitheodorou et al. 2021).

In a gradient of a chemoattractant, neutrophils are persistently polarized with a pseudopod oriented toward its source, which enables efficient directed migration (chemotaxis). On the other hand, when uniformly exposed to a chemoattractant, neutrophils polarize rapidly but transiently in a random direction. In both cases, GTP‐bound, active Cdc42 is enriched at the pseudopod to serve as a cell‐front coordinator (Yang, Collins, and Meyer 2016; Bell et al. 2021). The localized activation of Cdc42 has been demonstrated using Cdc42‐specific fluorescence resonance energy transfer (FRET) biosensors; however, it has remained obscure about the localization of endogenous Cdc42 in chemotaxis neutrophils.

For most migrating cells, localized actomyosin contractility is required at both the front and rear of the cell (Lawson and Ridley 2018; SenGupta, Parent, and Bear 2021); and protrusive Cdc42/Rac1 and contractile RhoA signals are both generated at the leading edge in a well‐coordinated manner (Nanda et al. 2023). Although this may be inconsistent with the prevailing idea that RhoA is excluded from the leading edge of migrating neutrophils (Wong et al. 2006; Yang, Collins, and Meyer 2016), periodic extension and retraction of pseudopods at the leading edge also occur in neutrophils (Zhelev, Alteraifi, and Chodniewicz 2004; Kamakura et al. 2013). In this oscillatory pseudopod formation, the extension is likely conducted by actin polymerization induced by Cdc42 and Rac; on the other hand, the retraction of protrusions may be mediated by myosin IIA contraction elicited by myosin light chain 2 (MLC2) phosphorylation, which can be promoted by the Rho‐associated protein kinase ROCK (Riento and Ridley 2003). Indeed, inhibition of RhoA or ROCK each results in a loss of pseudopod retraction in human neutrophils stimulated in a fMLP gradient (Zhelev, Alteraifi, and Chodniewicz 2004). Furthermore, in vivo experiments using a RhoA‐FRET mouse reveal that anterior and posterior fluctuation (oscillation) of RhoA activity occurs in neutrophils moving toward the site of tissue damage (Nobis et al. 2017), indicating that RhoA is periodically activated not only at the trailing edge but also at the leading edge of neutrophils. In addition, the RhoA effector mDia1 is recruited to the leading edge of chemotaxis neutrophils, thereby playing a crucial role in pseudopod formation and directed migration (Shi et al. 2009). These findings raise the possibility that RhoA also functions at the leading edge of neutrophils. However, the localization of endogenous RhoA in chemotaxis neutrophils has been largely unknown.

In spite of the significance, as described above, little is known about the localization of endogenous Cdc42 and RhoA in chemotaxis neutrophils. This may be partly due to the difficulty in immunochemical detection of endogenous Cdc42 and RhoA. For instance, usually‐used cell fixable agents, such as paraformaldehyde and methanol, are not suitable for the detection of endogenous Cdc42 and RhoA. However, in many cases, their visualization becomes possible when cells are fixed with trichloroacetic acid (TCA) (Higuchi et al. 2001; Yonemura, Hirao‐Minakuchi, and Nishimura 2004; Piekny, Werner, and Glotzer 2005; Yüce, Piekny, and Glotzer 2005; Kato et al. 2012; Hayase et al. 2013; see also Section 3).

In the present study, we show, by using a TCA fixation method, that endogenous RhoA, as well as endogenous Cdc42, is highly enriched at the pseudopod of human neutrophil‐like PLB‐985 cells that perceive C5a as uniform fields or gradients. The enrichment of RhoA is facilitated by Cdc42, indicating that Cdc42 serves as a positive regulator of RhoA at the front. ROCK1 is also recruited to the cell front during directed migration in a manner dependent on RhoA, Cdc42, and atypical protein kinase C (aPKC), an effector of Cdc42, thereby promoting phosphorylation of MLC2 at the pseudopod; on the other hand, ROCK1 localizes to the uropod when cells are uniformly stimulated. Thus, aPKC, as well as RhoA and Cdc42, appear to function together to recruit ROCK to the leading edge of chemotaxis neutrophils, thereby enhancing myosin II contractility for regulation of actomyosin dynamics at the front.

## 2 Results

### 2.1 Endogenous Cdc42 Localizes to the Pseudopod in Migrating Neutrophils for Correct Cell Polarization

**FIGURE 1:**
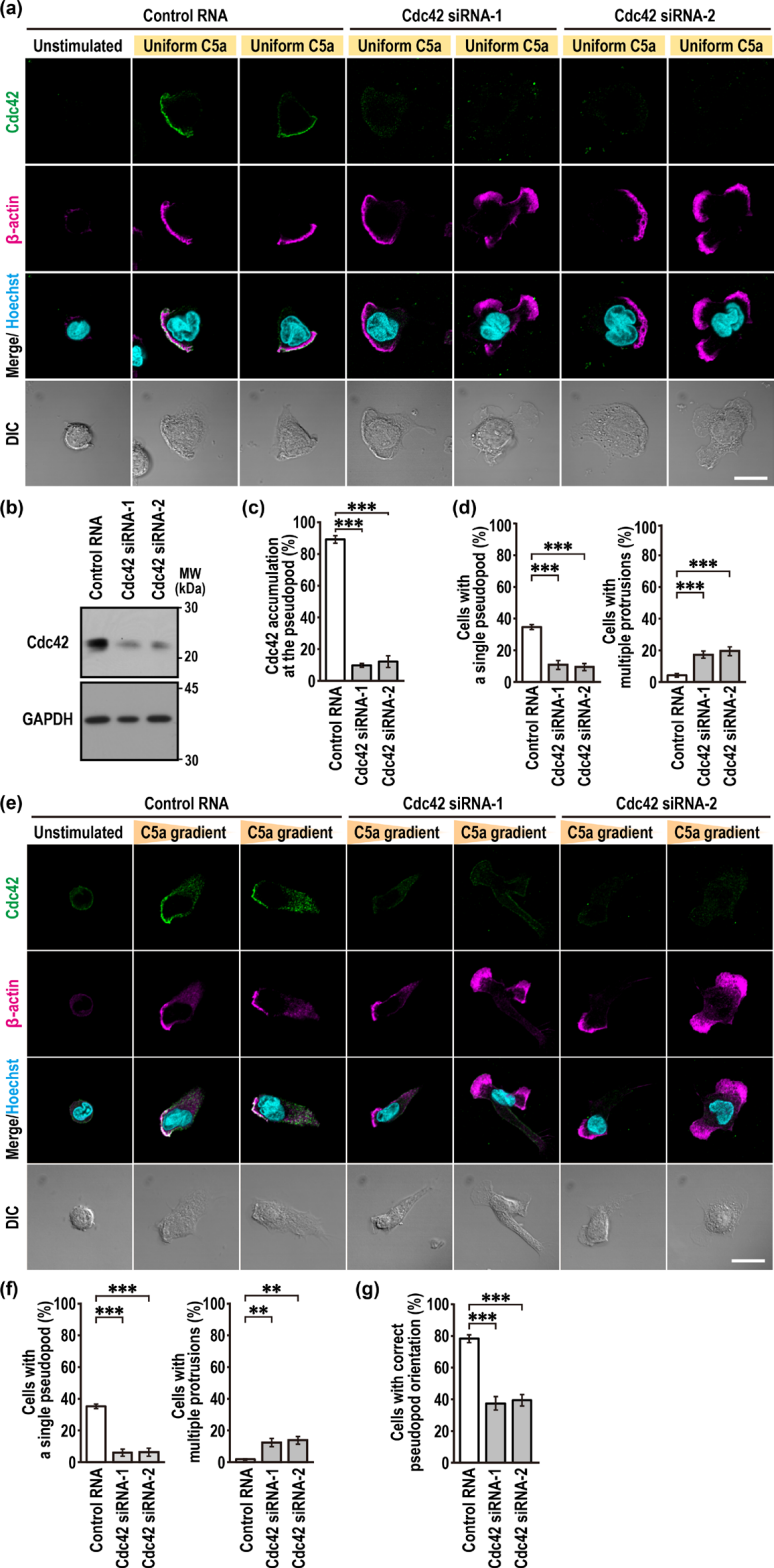
Endogenous Cdc42 localizes to the pseudopod in migrating neutrophils and participates in correct cell polarization. (a, e) Representative confocal images of PLB‐985 cells. Cells transfected with negative control RNA or Cdc42‐specific small interfering RNAs (siRNAs) (Cdc42 siRNA‐1 and siRNA‐2) were uniformly stimulated for 90 s with 3 nM C5a (a), or placed for 10 min in a Zigmond chamber with or without a C5a gradient (e). Cells were then fixed with 10% TCA and stained with the anti‐Cdc42 and anti‐β‐actin antibodies and Hoechst. The corresponding differential interference contrast (DIC) images are also shown. Scale bar = 10 μm. (b) Immunoblot analysis of Cdc42 siRNA‐transfected PLB‐985 cells with the anti‐Cdc42 and GAPDH antibodies. Positions for marker proteins are indicated in kDa. (c) Quantification of PLB‐985 cells with polarized accumulation of endogenous Cdc42 at the pseudopod induced by a uniform C5a stimulation. (d, f) Quantification of PLB‐985 cells with a single pseudopod (left) or those with multiple protrusions (right) when cells were stimulated uniformly with 3 nM C5a (d) or in a C5a gradient (f). (g) Quantification of PLB‐985 cells with a single pseudopod facing the source of C5a (correct pseudopod orientation). Values in c, d, f, and g are means ± SD from three independent experiments (*n* ≥ 100 cells/experiment). **p < 0.01 and ***p < 0.001 (Tukey–Kramer test).

Neutrophil stimulation with a uniformly applied chemoattractant leads to rapid but transient cell polarization, while the polarity persists in chemoattractant gradients (Iglesias and Devreotes 2008). Studies using FRET biosensors reveal that Cdc42 is activated during chemotaxis within the front of cells, including neutrophils (Yang, Collins, and Meyer 2016; Bell et al. 2021). However, localization of endogenous Cdc42 has not been studied except for a report demonstrating that it distributes within the front of mouse neutrophils in response to the chemotactic peptide fMLP (Kumar et al. 2012). To investigate localization of endogenous Cdc42 in human neutrophil‐like PLB‐985 cells (dimethyl sulfoxide [DMSO]‐differentiated PLB‐985 cells were used throughout the present study), we performed immunofluorescence staining using a specific monoclonal antibody after cell fixation with TCA, a protocol suitable for the use of this antibody (Higuchi et al. 2001; Hayase et al. 2013; see Section 4 and also Section 3). When uniformly stimulated with the complement‐derived chemoattractant C5a, PLB‐985 cells were polarized to form a single actin‐rich protrusion (a pseudopod) (Figure 1a), to which endogenous Cdc42 localized as confirmed by signal disappearance in its depleted cells (Figure 1b,c). In these experiments, we used an anti‐β‐actin antibody instead of phalloidin for actin staining because phalloidin did not work in TCA‐fixed samples. Depletion of Cdc42 resulted in a disturbed cell polarization with the formation of multiple (simultaneous) protrusions (Figure 1a,d), which is consistent with the previous finding that expression of dominant negative Cdc42 in human neutrophil‐like HL‐60 cells frequently induces the formation of multiple short‐lived leading edges that contain F‐actin when stimulated with a uniform concentration of fMLP (Srinivasan et al. 2003).

We next studied the localization of Cdc42 in PLB‐985 cells in a gradient of C5a in a Zigmond chamber. As shown in Figure 1e, chemotaxis cells acquired a polarized shape with an actin‐rich pseudopod facing the C5a gradient. Endogenous Cdc42 was accumulated to the leading edge during chemotaxis (Figure 1e). In Cdc42‐depleted cells, cell polarization was impaired with the formation of multiple protrusions (Figure 1e,f), which agrees with the previous observation that Cdc42‐knockout mouse neutrophils exhibit multiple protrusions in a gradient of fMLP (Szczur, Zheng, and Filippi 2009). The depletion also decreased in cells containing a single protrusion that was properly oriented toward the chemotactic source (Figure 1g), indicating an impaired directionality during chemotaxis of Cdc42‐depleted cells. Thus, endogenous Cdc42 likely localizes to the pseudopod in migrating neutrophils for proper cell polarization and directionality, whose localization is independent of whether or not a C5a gradient is present.

### 2.2 Endogenous RhoA Preferentially Localizes to the Pseudopod in Neutrophils Stimulated With a Uniform and a Gradient Concentration of C5a

**FIGURE 2:**
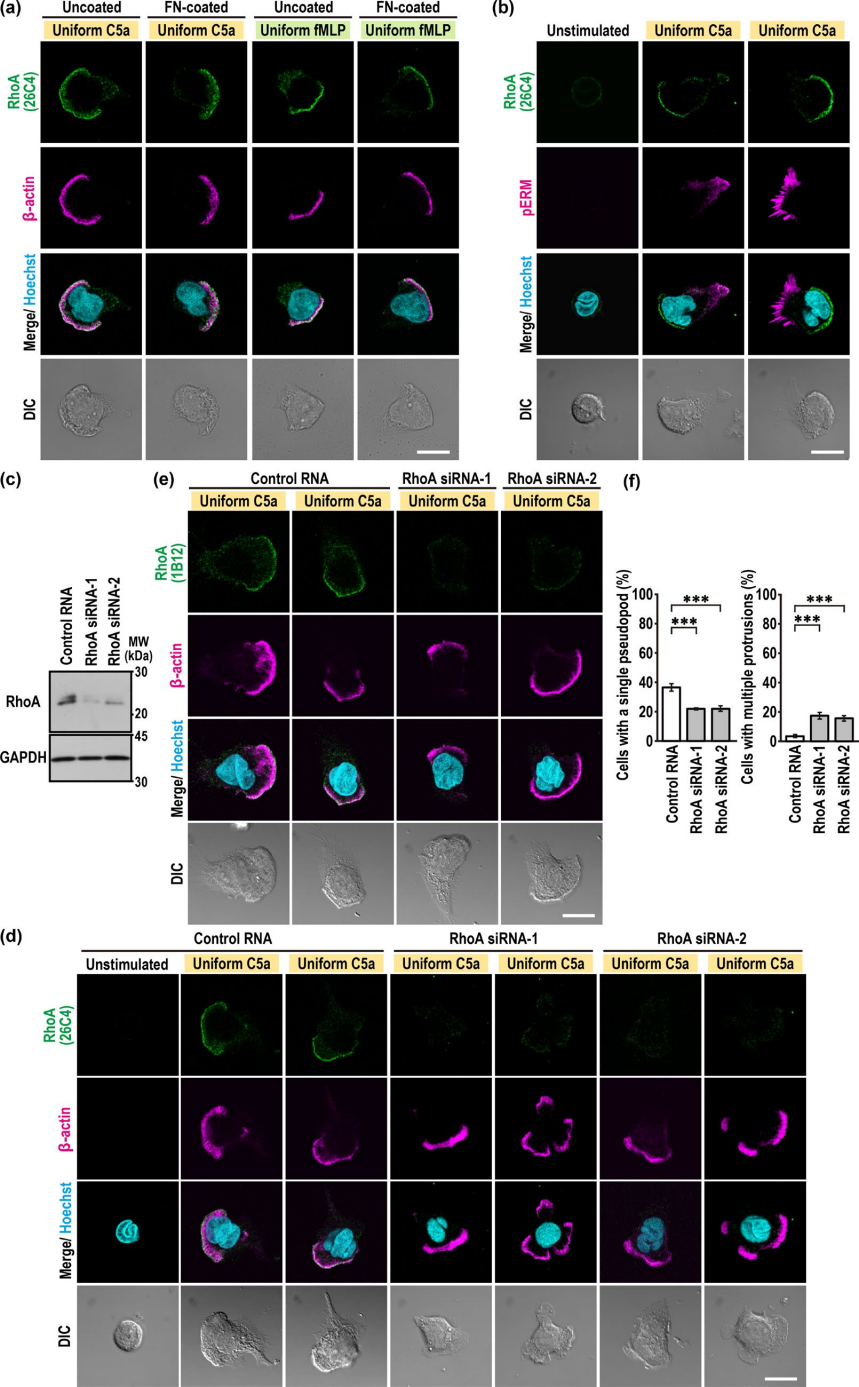
Endogenous RhoA preferentially localizes to the pseudopod in neutrophils stimulated with a uniform concentration of C5a. (a, b) Representative confocal images of PLB‐985 cells. Cells on glass surfaces without coating (uncoated) or coated with FN (FN‐coated) were uniformly stimulated for 90 s with 3 nM C5a or 100 nM fMLP as indicated. Cells were then fixed with 10% TCA and stained with the anti‐RhoA antibody (26C4), Hoechst, and the anti‐β‐actin or anti‐phospho‐ERM (pERM) antibody. The corresponding DIC images are also shown. (c) Immunoblot analysis of RhoA siRNA‐transfected PLB‐985 cells with the anti‐RhoA and GAPDH antibodies. Proteins in the lysates of PLB‐985 cells transfected with negative control RNA or RhoA‐specific siRNAs (RhoA siRNA‐1 and siRNA‐2) were analyzed by immunoblot with the anti‐RhoA (26C4) and anti‐GAPDH antibodies. Positions for marker proteins are indicated in kDa. (d, e) Representative confocal images of PLB‐985 cells stimulated with a uniform concentration of C5a. Cells were fixed with 10% TCA and stained with the anti‐RhoA monoclonal antibody clone 26C4 (d) or clone 1B12 (e). (f) Quantification of PLB‐985 cells with a single pseudopod (left) or multiple protrusions (right). Values are means ± SD from three independent experiments (*n* ≥ 100 cells/experiment). ***p < 0.001 (Tukey–Kramer test). Scale bar = 10 μm.

In addition to Cdc42, RhoA also contributes to polarization with a single pseudopod in migrating neutrophils (Xu et al. 2003; Yang, Collins, and Meyer 2016). It has been reported that in human neutrophil‐like HL‐60 cells (Xu et al. 2003) or mouse neutrophils (Li et al. 2005), each stimulated uniformly with fMLP, endogenous RhoA is generally excluded from pseudopods and distributed in the cytosol at the back and side (Xu et al. 2003; Li et al. 2005); in both cases, cells are fixed with paraformaldehyde before analysis by immunofluorescence microscopy. Further detailed analysis of endogenous RhoA, however, has not been performed. In particular, little is known about the distribution of endogenous RhoA during neutrophil chemotaxis in a chemoattractant gradient. To precisely analyze endogenous RhoA in migrating PLB‐985 cells, we used two distinct anti‐RhoA monoclonal antibodies (26C4 and 1B12) under an appropriate protocol including TCA fixation (Yonemura, Hirao‐Minakuchi, and Nishimura 2004; Piekny, Werner, and Glotzer 2005; Yüce, Piekny, and Glotzer 2005; Kato et al. 2012; see Section 4 and also Section 3). Analysis using the anti‐RhoA monoclonal antibody 26C4 showed that endogenous RhoA preferentially distributed to the pseudopod (Figure 2a) but not to the back region (Figure 2b) that is known to be abundant in phosphorylated‐ezrin/radixin/moesin (pERM) (Lacalle et al. 2007), in PLB‐985 cells stimulated uniformly with C5a. The accumulation of RhoA at the pseudopod was also induced when stimulated with fMLP instead of C5a (Figure 2a) and independent of the presence of fibronectin (FN), a component of the extracellular matrix (Figure 2a). As expected, the signal by 26C4 disappeared in RhoA‐depleted cells (Figure 2c,d). The preferential distribution of RhoA to the pseudopod was confirmed by the use of another anti‐RhoA monoclonal antibody, 1B12 (Figure 2e). Depletion of RhoA impaired cell polarization with the formation of multiple protrusions (Figure 2d,f), which is consistent with the previous finding that HL‐60 cells expressing dominant negative RhoA frequently form multiple protrusions when uniformly stimulated with fMLP (Xu et al. 2003).

**FIGURE 3:**
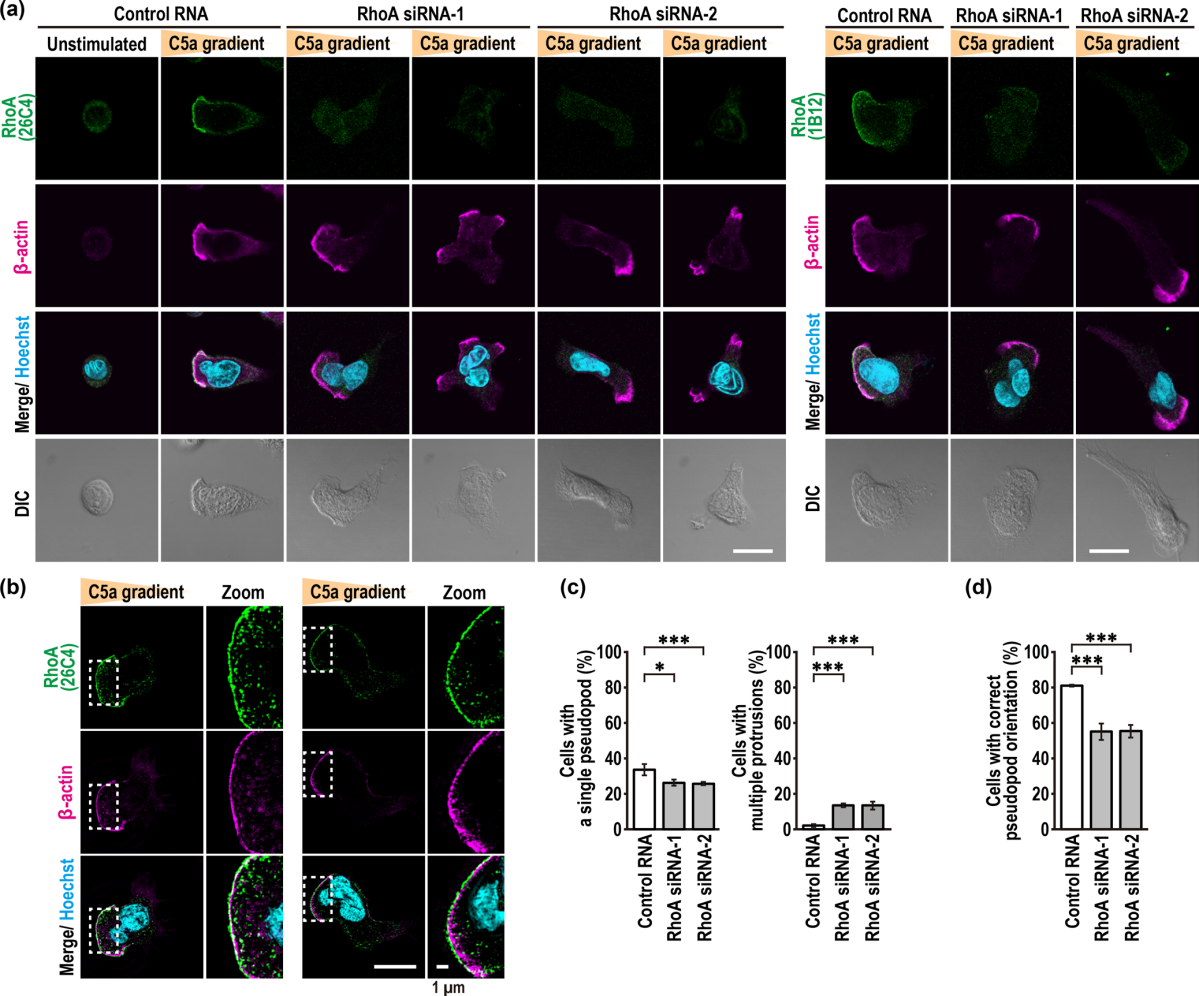
Endogenous RhoA preferentially localizes to the pseudopod of migrating neutrophils in a C5a gradient. (a) Representative confocal images of PLB‐985 cells in a C5a gradient. Cells transfected with negative control RNA or RhoA‐specific siRNAs (RhoA siRNA‐1 and siRNA‐2) were placed in a Zigmond chamber with or without a C5a gradient. Cells were then fixed with 10% TCA and stained with the anti‐RhoA monoclonal antibody clone 26C4 (left) or clone 1B12 (right), anti‐β‐actin antibody, and Hoechst. The corresponding DIC images are also shown. (b) Representative super‐resolution images of migrating PLB‐985 cells in a C5a gradient. The areas outlined with dashed lines are further magnified (zoom). (c, d) Quantification of PLB‐985 cells with a single pseudopod (left in c), with multiple protrusions (right in c), or with a single pseudopod facing the source of C5a (d). Values are means ± SD from three independent experiments (*n* ≥ 100 cells/experiment). *p < 0.05 and ***p < 0.001 (Tukey–Kramer test). The scale bars represent 10 μm, unless otherwise indicated.

Localization of endogenous RhoA during neutrophil chemotaxis in a chemoattractant gradient has not been reported. To detect endogenous RhoA in migrating PLB‐985 cells in a C5a gradient, we also used the two monoclonal antibodies 26C4 and 1B12 under the protocol involving TCA fixation. As shown in Figure 3a, endogenous RhoA was enriched at the leading edge of migrating cells in a C5a gradient. Super‐resolution imaging analysis by structured illumination microscopy (SIM) showed that RhoA was accumulated at the most anterior region of the actin‐rich pseudopod (Figure 3b), indicating its localization at the plasma membrane. Depletion of RhoA resulted in an increased formation of PLB‐985 cells with multiple protrusions (Figure 3a,c) and an impaired orientation of cells with a single protrusion (Figure 3d). Thus, endogenous RhoA is accumulated at the pseudopod during transient polarization induced by a uniform chemoattractant stimulation, in contrast to previous observations (Xu et al. 2003; Li et al. 2005); and endogenous RhoA is also enriched at the leading edge during persistent polarization in a chemoattractant gradient.

### 2.3 ROCK1 Distributes to the Rear of Neutrophils Polarized by Uniform C5a Stimulation in a Manner Independent of RhoA

**FIGURE 4:**
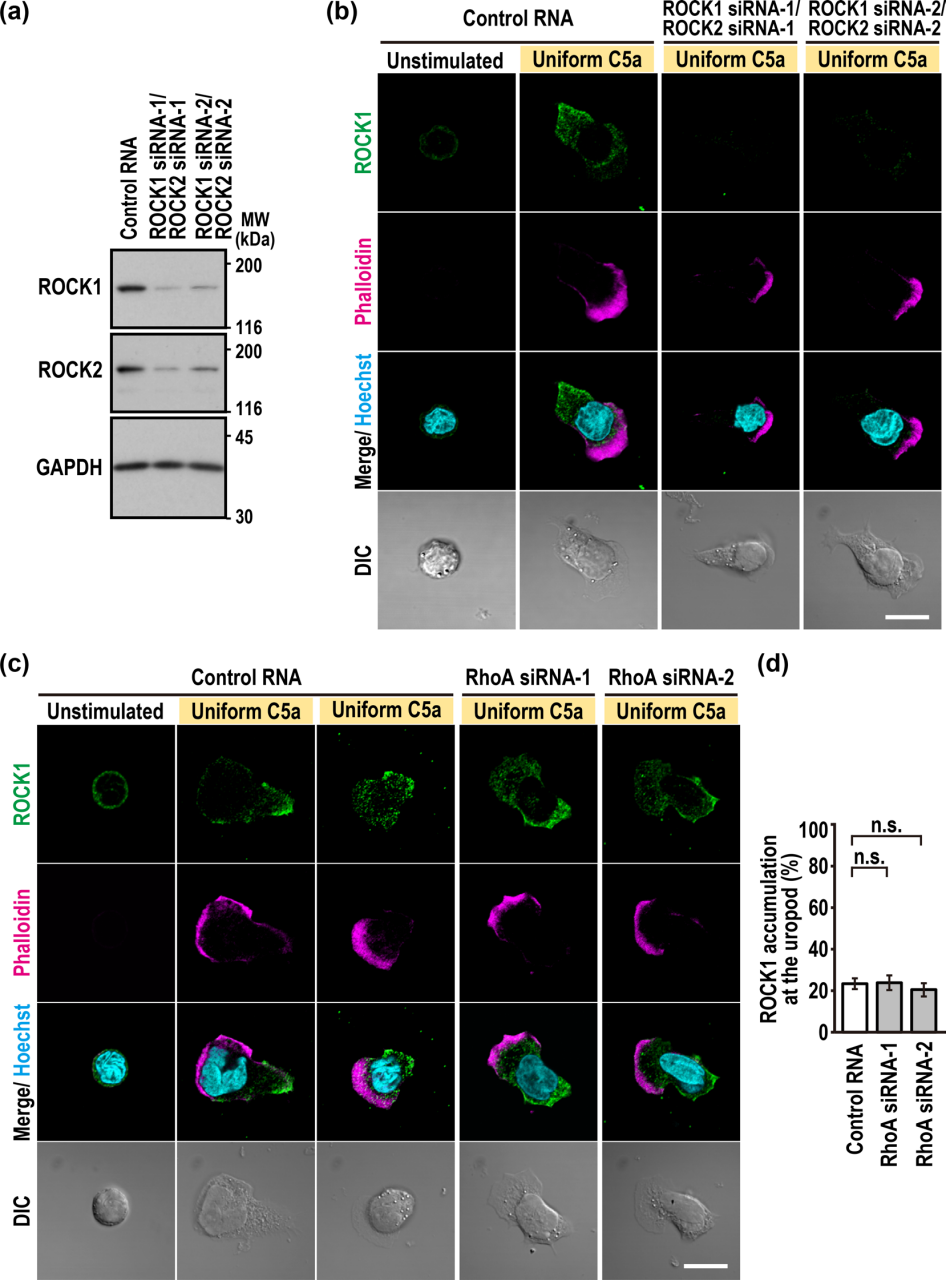
ROCK1 distributes to the rear of neutrophils polarized by uniform C5a stimulation in a manner independent of RhoA. (a) Immunoblot analysis of ROCK1/2 siRNA‐transfected PLB‐985 cells with the anti‐ROCK1, anti‐ROCK2, and anti‐GAPDH antibodies. Proteins in the lysates of PLB‐985 cells transfected with the indicated RNA were analyzed by immunoblot with the indicated antibodies. Positions for marker proteins are indicated in kDa. (b, c) Representative confocal images of PLB‐985 cells stimulated with 3 nM C5a. ROCK1/2‐depleted (b) or RhoA‐depleted (c) cells were fixed with 3.7% formaldehyde and stained with the anti‐ROCK1 antibody, phalloidin, and Hoechst. The corresponding DIC images are also shown. Scale bar = 10 μm. (d) Quantification of PLB‐985 cells with accumulation of endogenous ROCK1 at the uropod. Values are means ± SD from three independent experiments (*n* ≥ 100 cells/experiment). ns, not significant (Tukey–Kramer test).

Uniform stimulation of neutrophils with fMLP induces phosphorylation of MLC2 at the back (uropod) in polarized neutrophils (Xu et al. 2003; Shin et al. 2010) for myosin IIA‐mediated actomyosin contraction. Because the phosphorylation is blocked with Y‐27632 (Xu et al. 2003; Shin et al. 2010), a potent inhibitor of the Rho‐associated kinases ROCK1 and ROCK2 (Riento and Ridley 2003), ROCK is considered to regulate neutrophil migration by phosphorylating MLC2 at the uropod. It is, however, unknown about the distribution of endogenous ROCK1/2 in migrating neutrophils. Although human neutrophil‐like PLB‐985 cells expressed both ROCK1 and ROCK2, as indicated by immunoblot analysis with their specific antibodies (Figure 4a), we found that the anti‐ROCK1 but not anti‐ROCK2 antibody was suitable for immunofluorescence cell staining. As shown in Figure 4b, endogenous ROCK1 was distributed to the rear (the opposite side of the F‐actin‐rich pseudopod) in polarized PLB‐985 cells that were uniformly stimulated with C5a, which agrees with the idea that ROCK is expected to function at the uropod (Xu et al. 2003; Lacalle et al. 2007; Shin et al. 2010). Interestingly, depletion of RhoA did not affect the uropod localization of its effector ROCK1 (Figure 4c,d), which seems to be reasonable because RhoA was rather accumulated to the pseudopod in uniformly‐stimulated cells (Figure 2a,b).

### 2.4 ROCK1 Is Recruited to the Leading Edge of Migrating Neutrophils in a C5a Gradient, Which Involves RhoA and Cdc42

**FIGURE 5:**
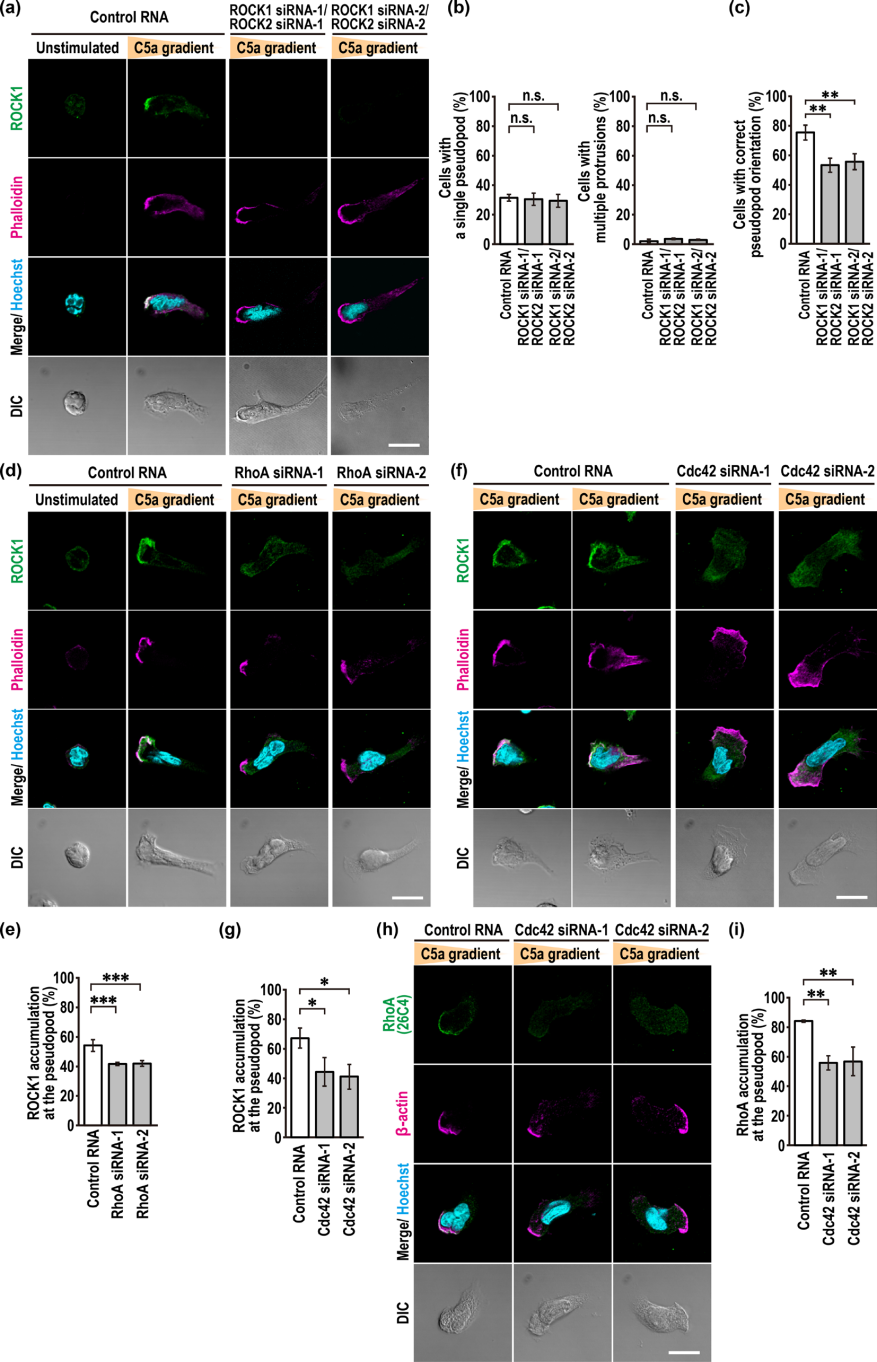
ROCK1 is recruited to the leading edge of migrating neutrophils in a C5a gradient, which involves RhoA and Cdc42. (a, d, f, h) Representative confocal images of PLB‐985 cells in a C5a gradient. Cells transfected with the indicated RNA were placed in a Zigmond chamber with or without a C5a gradient. Cells were then fixed with 3.7% formaldehyde (a, d, f) or 10% TCA (h) and stained as indicated. The corresponding DIC images are also shown. Scale bar = 10 μm. (b) Quantification of PLB‐985 cells with a single pseudopod (left) or multiple protrusions (right). (c) Quantification of cells with a single pseudopod facing the source of C5a (correct pseudopod orientation). (e, g, i) Quantification of PLB‐985 cells with accumulation of endogenous ROCK1 (e, g) or RhoA (i) at the pseudopod. Values are means ± SD from three independent experiments (*n* ≥ 100 cells/experiment). *p < 0.05; **p < 0.01; ***p < 0.001; and ns, not significant (Tukey–Kramer test).

Next, we investigated the localization of endogenous ROCK1 in neutrophil‐like PLB‐985 cells by chemotaxing them in a C5a gradient. In contrast to cells stimulated uniformly by C5a (Figure 4b), ROCK1 was accumulated to the F‐actin‐rich pseudopod in a C5a gradient (Figure 5a). Depletion of ROCK1/2 did not induce the formation of multiple protrusions (Figure 5b) but prevented correct pseudopod orientation (Figure 5c). Front accumulation of ROCK1 was partially but significantly inhibited by depletion of RhoA (Figure 5d,e), a GTPase that was also enriched to the pseudopod (Figure 3a,b). These findings suggest that endogenous ROCK1 is recruited to the leading edge for correct pseudopod orientation at least partially via its interaction with RhoA.

Since Cdc42, as well as RhoA, was accumulated to the leading edge during chemotaxis (Figure 1e), we tested its role in the recruitment of ROCK1. Depletion of Cdc42 in PLB‐985 cells substantially reduced the localization of endogenous ROCK1 to the pseudopod during chemotaxis in a gradient of C5a (Figure 5f,g). Thus, Cdc42 also appears to contribute to cell‐front recruitment of ROCK1. Intriguingly, RhoA localization to the pseudopod was impaired in Cdc42‐depleted PLB‐985 cells (Figure 5h,i); this effect, that is, the Cdc42‐enhanced RhoA accumulation, may be explicable from the ability of Cdc42 to activate RhoA, as shown in neutrophil‐like HL‐60 cells (Van Keymeulen et al. 2006). Taken together, it seems likely that Cdc42 enhances the accumulation of RhoA to the pseudopod, which leads to an increase in RhoA‐dependent recruitment of ROCK1.

### 2.5 The Cdc42‐Downstream Kinase aPKC Enhances Cell Front Recruitment of ROCK and Plays a Crucial Role in Neutrophil Chemotaxis

**FIGURE 6:**
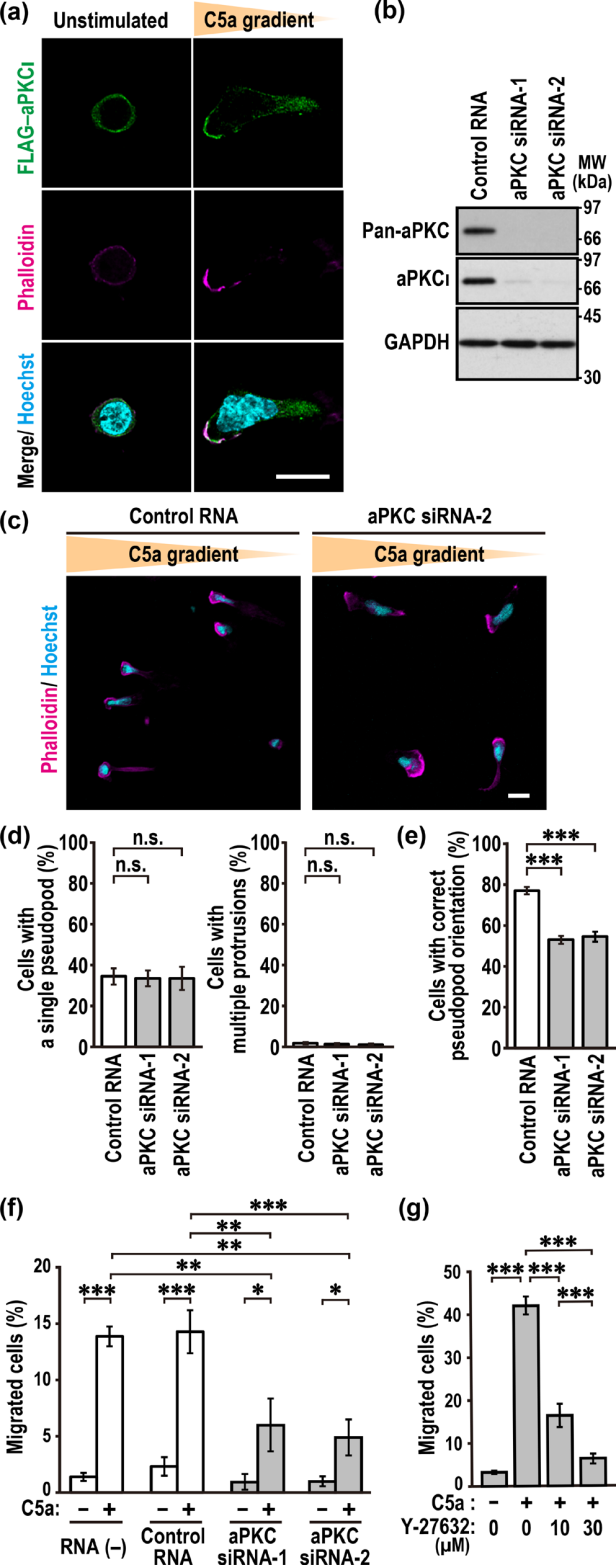
The Cdc42‐downstream kinase aPKC participates in C5a‐induced neutrophil chemotaxis. (a) Representative confocal images of PLB‐985 cells expressing FLAG–aPKCι. Cells transfected with FLAG‐tagged aPKCι were placed in a Zigmond chamber with or without a C5a gradient. Cells were then fixed with 3.7% formaldehyde and stained with the anti‐FLAG antibody, phalloidin, and Hoechst. (b) Immunoblot analysis of aPKC siRNA‐transfected PLB‐985 cells with the anti‐aPKC and GAPDH antibodies. Proteins in the lysates of PLB‐985 cells transfected with negative control RNA or aPKC‐specific siRNAs (aPKC siRNA‐1 and siRNA‐2) were analyzed by immunoblot with the anti‐pan‐aPKC, anti‐aPKCι, and anti‐GAPDH antibodies. Positions for marker proteins are indicated in kDa. (c) Representative confocal images of aPKC‐depleted PLB‐985 cells in a C5a gradient. Cells were fixed with 3.7% formaldehyde and stained with phalloidin and Hoechst. (d, e) Quantification of aPKC‐depleted PLB‐985 cells with a single pseudopod (left in d), with multiple protrusions (right in d), or with a single pseudopod facing the source of C5a (e). Values are means ± SD from three independent experiments (*n* ≥ 100 cells/experiment). (f, g) Chemotactic response of PLB‐985 cells. Migration of cells transfected with the indicated RNA (f) or cells treated with Y‐27632 (g) in response to C5a was measured in transwell chemotaxis chambers. The percentage of migrated cells was calculated by dividing the number of migrated cells by that of the input cells. Values are means ± SD from three independent experiments. *p < 0.05; **p < 0.01; ***p < 0.001; and ns, not significant (Tukey–Kramer test). Scale bar = 10 μm.

The Cdc42‐dependent recruitment of ROCK to the leading edge (Figure 5f,g) may be induced in not only RhoA‐dependent but also RhoA‐independent manner because the recruitment seemed to be more efficiently inhibited by Cdc42 depletion (Figure 5g) compared with the inhibition by RhoA knockdown (Figure 5e). The Cdc42‐interacting protein kinase aPKC, which forms a stable complex with Par6 to associate with GTP‐bound Cdc42 (Hayase et al. 2013), is a possible candidate for a downstream effector of Cdc42 in cell‐front recruitment of ROCK during chemotaxis. In this context, it should be noted that aPKC is accumulated to the pseudopod in migrating mouse neutrophils to promote correct pseudopod orientation, thereby playing a crucial role in chemotaxis (Kamakura et al. 2013). Hence, we next investigated the role of aPKC during the chemotaxis of human neutrophil‐like PLB‐985 cells. As shown in Figure 6a, during cell migration in a gradient of C5a, FLAG‐tagged aPKCι was recruited to the pseudopod; unfortunately, however, it remained unknown endogenous aPKC in PLB‐985 cells because it was not efficiently stained even with the anti‐aPKC antibody suitable for staining of aPKC in mouse neutrophils (Kamakura et al. 2013). Depletion of aPKC in PLB‐985 cells (Figure 6b) did not affect the ability to polarize in a C5a gradient (Figure 6c,d) but impaired the correct orientation of the pseudopod (Figure 6c,e). Consistent with this, aPKC‐depleted cells failed to efficiently migrate toward C5a (Figure 6f) under the conditions where chemotaxis was blocked by Y‐27632 (Figure 6g), a ROCK inhibitor that is known to prevent neutrophil chemotaxis in a fMLP gradient (Alblas et al. 2001). Thus, aPKC is recruited to the leading edge to play a crucial role in the chemotaxis of human PLB‐985 cells.

**FIGURE 7:**
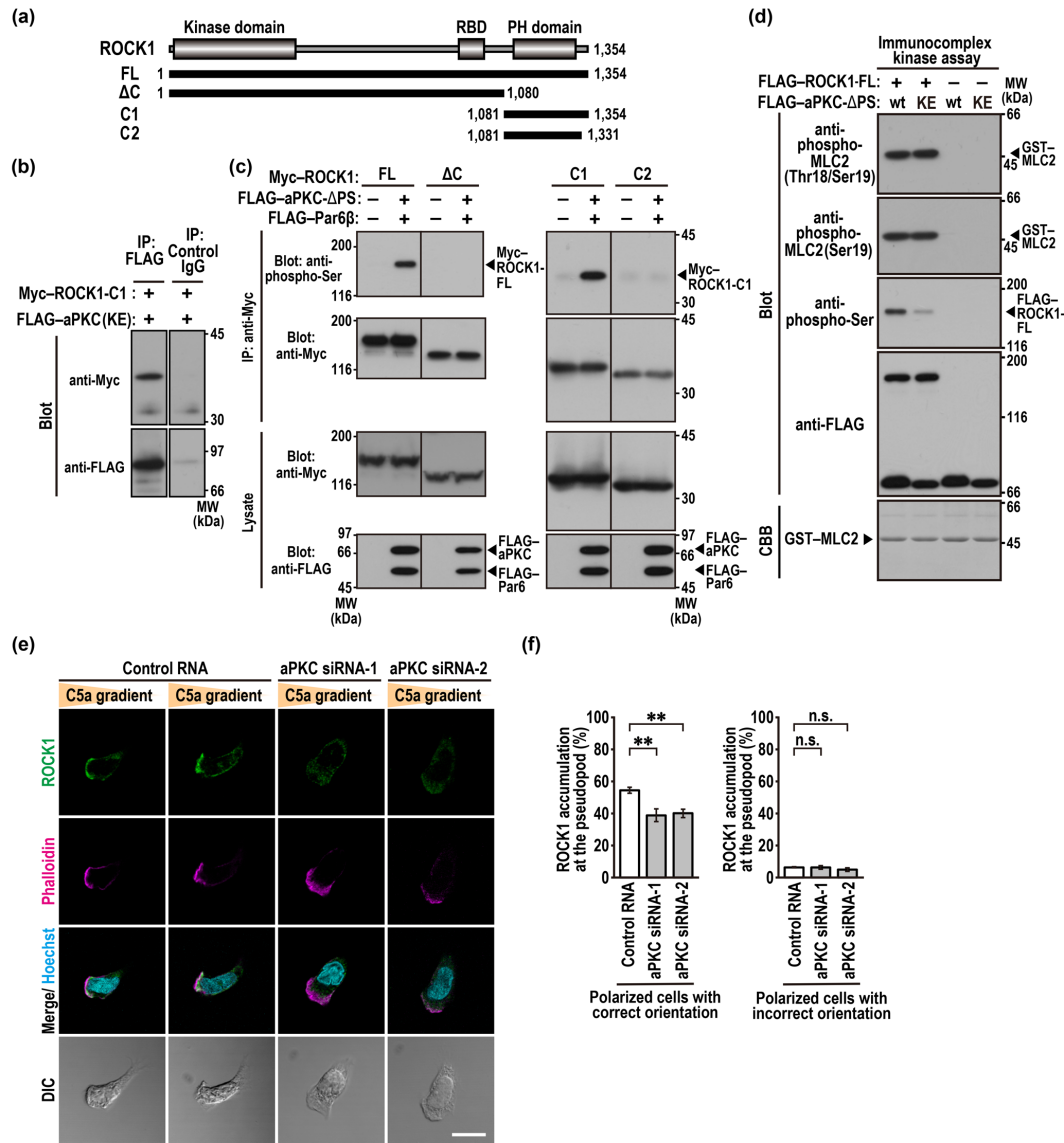
aPKC is capable of interacting with ROCK and involved in recruitment of ROCK1 to the leading edge. (a) Schematic representation of constructs for full length (FL) and truncated forms (ΔC, C1, and C2) of human ROCK1. RBD, Rho‐binding domain; and PH, pleckstrin homology. (b) Interaction between aPKCι and the C‐terminal region of ROCK1. FLAG–aPKCι (K274E) was co‐expressed with Myc–ROCK1‐C1 in HEK293 cells, and proteins in the cell lysate were immunoprecipitated (IP) with the anti‐FLAG M2 monoclonal antibody or control IgG, followed by immunoblot analysis with the indicated antibodies. Positions for marker proteins are indicated in kDa. (c) aPKC‐mediated phosphorylation of ROCK1. FLAG–aPKCι‐ΔPS (wt) and FLAG–Par6β were co‐expressed with various lengths of Myc–ROCK1 in HEK293 cells, and proteins in the cell lysate were immunoprecipitated (IP) with the anti‐Myc monoclonal antibody, followed by immunoblot analysis with the anti‐Myc, anti‐FLAG, and anti‐phospho‐Ser‐PKC substrate antibodies. (d) The effect of aPKC‐mediated phosphorylation of ROCK1 on the catalytic activity of ROCK1. FLAG–ROCK1‐FL and FLAG–aPKCι‐ΔPS (wt or K274E) were separately purified by immunoprecipitation from HEK293 cells expressing either FLAG‐tagged protein. The two purified proteins were mixed and used for measuring ROCK1 activity using GST–MLC2 as a substrate by an immunocomplex kinase assay, as described in Section 4. Phosphorylation of GST–MLC2 was analyzed by immunoblot with the anti‐phospho‐MLC2 antibodies (Thr18/Ser19 or Ser19); and the aPKC‐mediated phosphorylation of ROCK1 was also observed with the anti‐phospho‐Ser‐PKC substrate antibody. The amounts of FLAG and GST proteins in each reaction mixture were determined by immunoblot with the anti‐FLAG antibody and staining with CBB, respectively. (e) Representative confocal images of PLB‐985 cells in a C5a gradient. Cells transfected with the indicated RNA were placed in a Zigmond chamber with a C5a gradient. Cells were then fixed with 3.7% formaldehyde and stained as indicated. The corresponding DIC images are also shown. Scale bar = 10 μm. (f) Effect of aPKC depletion on accumulation of endogenous ROCK1 at the pseudopod of PLB‐985 cells in a C5a gradient. The graphs show the percentage of cells with a ROCK1‐accmulated pseudopod in the total of cells with a single, correctly‐oriented pseudopod (left) or in the total of cells with a single, incorrectly oriented pseudopod (right). Values are means ± SD from three independent experiments (*n* ≥ 100 cells/experiment). **p < 0.01; and ns, not significant (Tukey–Kramer test).

To know the role of the Cdc42‐binding protein aPKC in the Cdc42‐dependent ROCK recruitment to the leading edge (Figure 5f,g), we tested the possibility that aPKC also interacts with ROCK, since it has been reported that aPKCι, an isoform of aPKC, can phosphorylate the C‐terminal region of ROCK1 (Ishiuchi and Takeichi 2011). For this purpose, we expressed a catalytically inactive form of aPKCι (K274E) in HEK293 cells and analyzed its co‐precipitated proteins by liquid chromatography (LV)–tandem mass spectrometry (MS/MS) (see Section 4). The analysis identified ROCK1 as an aPKC‐interacting protein. Furthermore, immunoblot analysis of precipitated proteins revealed that aPKCι bound to the C‐terminal region of ROCK1 (Figure 7a,b). By expressing a constitutively active aPKCι (aPKC‐ΔPS) and its partner protein Par6β (Figure 7c), we confirmed the ability of aPKCι to phosphorylate the C‐terminus of ROCK1 (Ishiuchi and Takeichi 2011). On the other hand, aPKC‐mediated phosphorylation did not affect the catalytic activity of ROCK1, as shown by an in vitro kinase assay using glutathione *S*‐transferase (GST)‐tagged MLC2 as a substrate of ROCK1 in the presence of active aPKCι (Figure 7d). These findings indicate that aPKC interacts with and phosphorylates ROCK1, but the phosphorylation does not activate ROCK1.

**FIGURE 8:**
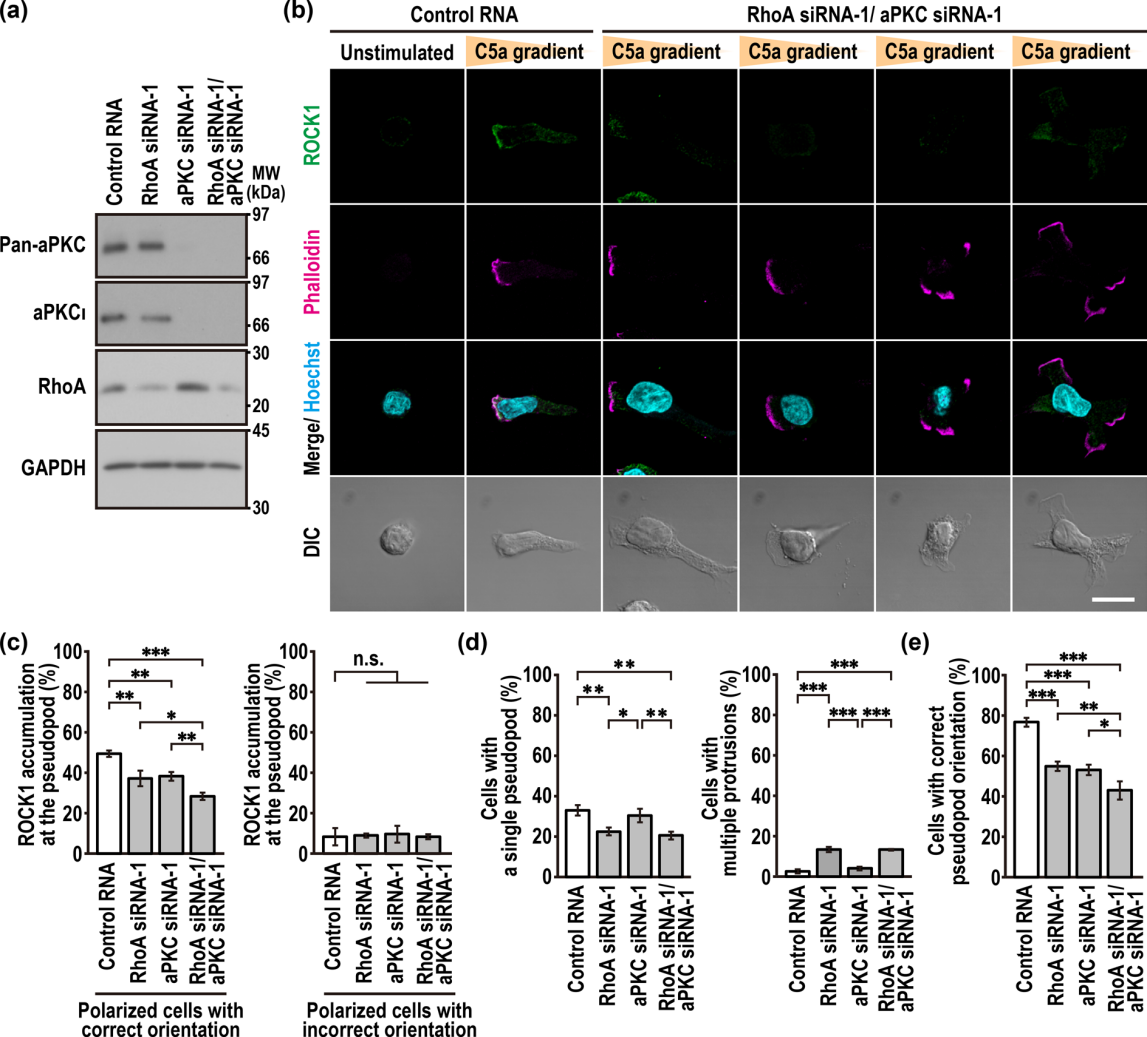
aPKC and RhoA cooperatively recruit ROCK to the leading edge for efficient chemotaxis. (a) Immunoblot analysis of aPKC and RhoA expression in PLB‐985 cells transfected with the indicated RNA. Positions for marker proteins are indicated in kDa. (b) Representative confocal images of PLB‐985 cells in a C5a gradient. Cells transfected with the indicated RNA were placed in a Zigmond chamber with a C5a gradient. Cells were then fixed with 3.7% formaldehyde and stained as indicated. The corresponding DIC images are also shown. Scale bar = 10 μm. (c) Effect of RhoA and aPKC depletion on accumulation of endogenous ROCK1 at the pseudopod of PLB‐985 cells in a C5a gradient. The graphs show the percentage of cells with a ROCK1‐accmulated pseudopod in the total of cells with a single, correctly‐oriented pseudopod (left) or in the total of cells with a single, incorrectly oriented pseudopod (right). (d, e) Quantification of PLB‐985 cells with a single pseudopod (left in d), with multiple protrusions (right in d), or with a single pseudopod facing the source of C5a (e). Values in c–e are means ± SD from three independent experiments (*n* ≥ 100 cells/experiment). *p < 0.05; **p < 0.01; ***p < 0.001; and ns, not significant (Tukey–Kramer test).

We next investigated the role of the Cdc42‐binding protein aPKC in ROCK1 recruitment to the pseudopod in chemotaxis PLB‐985 cells. Depletion of aPKC significantly prevented ROCK1 from accumulating at the leading edge (Figure 7e,f), which indicates that aPKC contributes to ROCK1 recruitment. Taken together with the present findings that front accumulation of ROCK1 also involves RhoA (Figure 5d,e) and, more significantly Cdc42 (Figure 5f,g), it seems possible that Cdc42 promotes ROCK recruitment to the pseudopod via the Cdc42‐binding protein aPKC and RhoA, the latter of which is shown to be enriched at the leading edge in a Cdc42‐dependent manner (Figure 5h,i). Consistent with the idea, the impairment of ROCK recruitment by depletion of RhoA was enhanced by simultaneous knockdown of aPKC (Figure 8a–c); and vice versa (Figure 8a–c). On the other hand, the formation of multiple pseudopods was induced by the depletion of RhoA in a manner independent of aPKC knockdown (Figure 8b,d). Thus, although RhoA but not aPKC participates in cell polarization with a single pseudopod, the two proteins appear to serve together to recruit ROCK to the leading edge, thereby leading to correct pseudopod orientation (Figure 8e) for efficient chemotaxis.

### 2.6 ROCK Promotes MLC Phosphorylation at the Leading Edge During Neutrophil Chemotaxis in a Gradient of C5a

**FIGURE 9:**
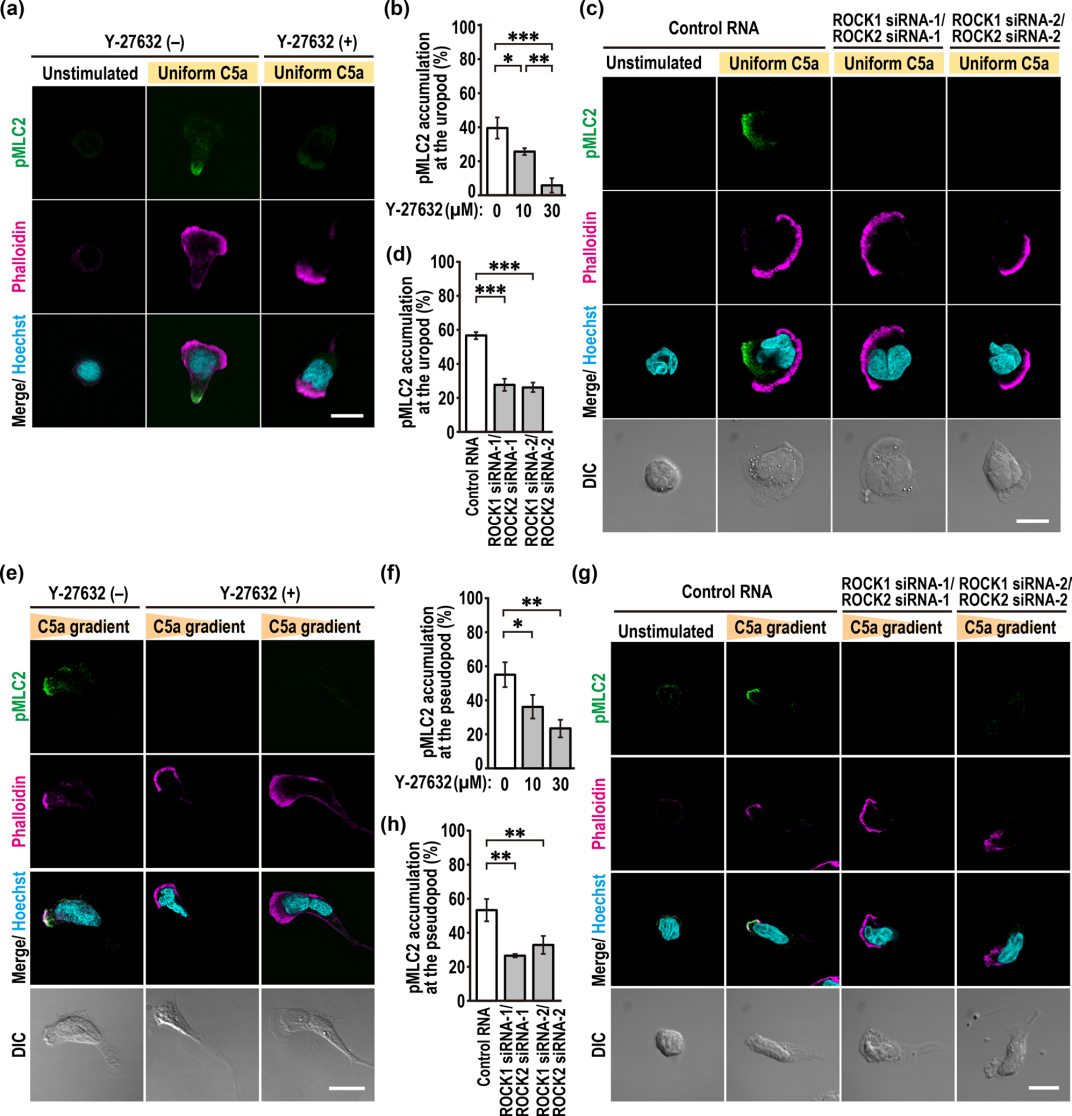
ROCK phosphorylates MLC at the leading edge during neutrophil chemotaxis in a gradient of C5a. (a, e) Representative confocal images of PLB‐985 cells treated with Y27632. Cells were uniformly stimulated with or without 3 nM C5a (a), or placed in a Zigmond chamber with a C5a gradient (e) in the absence (−) or presence (+) of 10 μM Y27632. Cells were then fixed with 3.7% formaldehyde and stained with the anti‐pMLC2 (Ser19) antibody, phalloidin, and Hoechst. (b, d) Quantification of Y27632‐treated (b) or ROCK1/2‐depleted (d) PLB‐985 cells with uropod accumulation of pMLC2 induced by a uniform concentration of C5a. (c, g) Representative confocal images of PLB‐985 cells. Cells transfected with the indicated RNA were uniformly stimulated for 90 s with 3 nM C5a (c), or placed in a Zigmond chamber with or without a C5a gradient (g). Cells were then fixed with 3.7% formaldehyde and stained with the indicated antibodies and Hoechst. The corresponding DIC images are also shown. (f, h) Quantification of Y27632‐treated (f) or ROCK1/2‐depleted (h) PLB‐985 cells with pseudopod accumulation of pMLC2 in a C5a gradient. Values in b, d, f, and h are means ± SD from three independent experiments (*n* ≥ 100 cells/experiment). *p < 0.05; **p < 0.01; and ***p < 0.001 (Tukey–Kramer test). Scale bar = 10 μm.

Proper neutrophil chemotaxis requires non‐muscle myosin IIA‐dependent tractions at both the leading and the trailing edge; traction development requires phosphorylation of the regulatory protein MLC2 (Shin et al. 2010). In neutrophils uniformly stimulated with fMLP, phosphorylated MLC2 (pMLC2) is highly enriched at the uropod, which is inhibited by Y‐27632 (Xu et al. 2003; Shin et al. 2010). ROCK‐mediated phosphorylation of MLC2 at the uropod was also observed in PLB‐985 cells exposed to a uniform concentration of C5a, as shown by a reduced appearance of pMLC2 not only in Y‐27632‐treated cells (Figure 9a,b) but also in ROCK1/2‐depleted cells (Figure 9c,d). The crucial role of ROCK in the phosphorylation of MLC2 at the uropod is in accordance with the accumulation of endogenous ROCK1 to the rear region in transiently polarized PLB‐985 cells (Figure 4b).

On the other hand, little is known about the phosphorylation of endogenous MLC2 during neutrophil chemotaxis in a chemoattractant gradient, although it is generally thought that the phosphorylation dominantly occurs at the rear, where myosin IIA is enriched. In contrast to uniformly stimulated PLB‐985 cells, endogenous MLC2 was phosphorylated at the leading edge during chemotaxis in a C5a gradient (Figure 9e). Pseudopod localization of pMLC2 may not be surprising because substantial amounts of myosin IIA are distributed at the front of chemotaxis neutrophils (Shin et al. 2010; Hadjitheodorou et al. 2021). The phosphorylation was efficiently blocked by cell treatment with Y‐27632 (Figure 9e,f) and by depletion of ROCK1/2 (Figure 9g,h). Thus, ROCK appears to promote phosphorylation of MLC at the pseudopod to develop tractions required for proper neutrophil chemotaxis.

## 3 Discussion

In the present study, we show that endogenous RhoA (Figures 2a,b,d,e and 3a,b), as well as endogenous Cdc42 (Figure 1a,e), accumulates to the pseudopod of neutrophil‐like PLB‐985 cells in response to C5a, by using their respective specific antibodies after fixation with TCA. Imaging that relies on cell fixation is not suited for studying dynamic cellular events because of poor temporal resolution, but is required for exploring precise localization of endogenous proteins. TCA fixation may be beneficial for a certain type of antibodies because TCA makes hidden epitopes more accessible via its denaturing effect (Martin et al. 2022; Echeverria Jr, Leathers, and Rogers 2025), whereas formaldehyde fixation is convenient for targeting structural epitopes (Stumptner et al. 2019). In addition to this possible benefit, TCA fixation seems to be suitable for the detection of an active form of Rho‐family small GTPases, such as RhoA and Cdc42, for the following reasons. The vast majority of RhoA, as well as Cdc42 in cells, is in a GDP‐bound state and stably dimerized with RhoGDI in the cytosol (Garcia‐Mata, Boulter, and Burridge 2011); as a result, RhoA is found diffusely located throughout the cytoplasm, which seems to obscure the visualization of an active form of endogenous RhoA under formaldehyde fixation (Piekny, Werner, and Glotzer 2005). In addition, compared with Rac and Cdc42, RhoA has a more tendency to reside in the cytosol when overexpressed in cells (de Seze, Gatin, and Coppey 2023). The cytosolic pool of RhoA is efficiently extracted by TCA fixation but not by the use of crosslinking fixatives such as formaldehyde (Piekny, Werner, and Glotzer 2005; Koh, Pham, and Piekny 2022). Indeed, TCA‐fixed RhoA is predominantly detected at a specific domain of the plasma membrane in a variety of cell types (Yonemura, Hirao‐Minakuchi, and Nishimura 2004; Yüce, Piekny, and Glotzer 2005; Kato et al. 2012); also in neutrophil‐like PLB‐985 cells, RhoA is preferentially accumulated at the pseudopod (Figures 2a,b,d,e and 3a) and its localization to the plasma membrane is further confirmed by super‐resolution microscopic analysis (Figure 3b). Since active RhoA is associated with membranes (Garcia‐Mata, Boulter, and Burridge 2011), TCA‐fixed RhoA appears to be in an active state (Piekny, Werner, and Glotzer 2005; Koh, Pham, and Piekny 2022). Due to the same reason for RhoA, TCA‐fixed Cdc42 is detected at a specific domain of the plasma membrane, probably in an active form, in various types of cells (Higuchi et al. 2001; Hayase et al. 2013) as in PLB‐985 cells (Figure 1a,e).

The accumulation of endogenous, active Cdc42 at the pseudopod of chemotaxis neutrophils (Figure 1e) seems to be in keeping with thefront localization of FRET‐based biosensors for the detection of Cdc42 activation (Yang, Collins, and Meyer 2016; Bell et al. 2021). On the other hand, in contrast to the present finding of pseudopod localization of active RhoA, active (GTP‐bound) RhoA is considered to be excluded from the leading edge in chemotaxis neutrophils on the basis of the analysis using RhoA‐FRET biosensors (Wong et al. 2006; Yang, Collins, and Meyer 2016). The reason for this discrepancy is presently unknown. In many cell types except neutrophils, FRET microscopic analysis has shown that RhoA is activated at both the leading edge and trailing end during cell migration (Lawson and Ridley 2018; SenGupta, Parent, and Bear 2021; Nanda et al. 2023); and localized actomyosin contractility is also required at both the front and rear of the cells (Lawson and Ridley 2018). The present finding of enrichment of active RhoA at the front of chemotaxis neutrophils (Figure 3a,b) may explain well the reason why the following events occur: blockade of pseudopod retraction at the leading edge by RhoA and ROCK inhibitors (Zhelev, Alteraifi, and Chodniewicz 2004), and recruitment of the RhoA effector mDia1 to the pseudopod (Shi et al. 2009). Furthermore, in vivo experiments using a RhoA‐FRET mouse reveal that anterior and posterior fluctuation (oscillation) of RhoA activity occurs in neutrophils moving toward the site of tissue damage (Nobis et al. 2017).

At the leading edge, RhoA may serve as a positive and negative regulator of pseudopod formation. RhoA appears to contribute to the formation of a single pseudopod, as indicated by the present finding that depletion of RhoA induces the formation of multiple pseudopods (Figures 2 and 3). This effect may be mediated via the RhoA effector mDia1, an actin polymerizing protein that is recruited to the front and crucial for pseudopod formation (Shi et al. 2009). On the other hand, ROCK, another major effector of RhoA, promotes phosphorylation of MLC2 at the front (Figure 9), probably inducing myosin IIA‐mediated retraction of the pseudopod (Zhelev, Alteraifi, and Chodniewicz 2004); the retraction may destabilize the pseudopod at the front, which destabilization is considered to suppress cell directionality during chemotaxis (Kamakura et al. 2013). Thus, ROCK can negatively regulate chemotaxis. In this context, it seems interesting that deficiency of ROCK1 results in increased migration of neutrophils both in vivo and in vitro (Vemula et al. 2010). The opposite function of the two major RhoA effectors, mDia and ROCK, on pseudopod dynamics seems to allow RhoA to fine‐tune neutrophil chemotaxis. Intriguingly, deletion of RhoA leads to upregulation or downregulation of neutrophil chemotaxis, depending on the type of chemoattractants used (Jennings et al. 2014).

In contrast to the ability of RhoA and Cdc42 to maintain a single protrusion (Figures 1, 2, 3), aPKC appears to regulate chemotaxis in a distinct process. Depletion of aPKC suppresses the correct pseudopod orientation along a C5a gradient (Figure 6e) without promoting the formation of multiple protrusions (Figure 6d), leading to an impairment of directed neutrophil migration (Figure 6f). These observations support the idea that aPKC is involved in directionality control during chemotaxis but not in cell polarization. The involvement has been originally and specifically shown in a previous study, in which various aPKC inhibitors all do prevent directional movement but not suppress the moving velocity during mouse neutrophil chemotaxis in a gradient of fMLP (Kamakura et al. 2013).

Recruitment of aPKC to the leading edge is possibly mediated by active Cdc42, which potently interacts with Par6, a stable binding partner of aPKC (Hayase et al. 2013). GTP‐bound Cdc42 at the front is known to control the steering of polarized neutrophils (Yang, Collins, and Meyer 2016), which may be mediated by the directionality regulator aPKC. In addition to Cdc42, aPKC enrichment at the pseudopod may also involve the polarity proteins mInsc and Par3. This is because chemoattractant receptor‐mediated targeting of mInsc to the front of migrating neutrophils leads to recruitment of Par6–aPKC via Par3, an adaptor protein capable of associating simultaneously with mInsc and Par6–aPKC (Kamakura et al. 2013). The present study also indicates that aPKC regulates cell directionality partially via the recruitment of ROCK. aPKC interacts with ROCK (Figure 7b) and contributes to ROCK recruitment to the leading edge (Figure 7e,f); and ROCK participates in pseudopod orientation but not in cell polarization (Figure 5a–c) as does aPKC (Figure 6c–e). Thus, ROCK appears to not only drive actomyosin contractility at the pseudopod (Figure 9) but also regulate pseudopod orientation (Figure 5); however, the molecular link between the two events remains to be elucidated.

It is known that active Cdc42 at the leading edge activates its effector WASP to form an actin filament‐based protrusion as a motive force (Kumar et al. 2012; Brunetti et al. 2022). The present study suggests that Cdc42 also promotes the recruitment of ROCK1 to the pseudopod of neutrophils with persistent polarity during chemotaxis in a C5a gradient (Figure 5f,g). This effect seems to be mediated via not only the Cdc42‐interacting protein aPKC but also RhoA, which localizes to the front in a Cdc42‐dependent manner (Figure 5h,i). In contrast, when stimulated uniformly with C5a, ROCK1 is accumulated to the uropod of transiently‐polarized neutrophils (Figure 4b–d) in a manner independent of RhoA (Figure 4c,d). Thus, endogenous ROCK1 is differently recruited between persistently and transiently polarized cells. Consistent with this, ROCK‐mediated MLC2 phosphorylation, an indicator of actomyosin contraction, occurs mainly at the pseudopod in chemotaxis neutrophils (Figure 9e–h) but at the uropod in transiently‐polarized cells (Figure 9a–d). Taken together with the present findings, contractile actomyosin dynamics and actin network formation are both induced predominantly at the leading edge (pseudopod) of neutrophils migrating in a chemoattractant gradient. The cell‐front coordinator Cdc42 and Rac1 cooperate to form protrusions at the front by promoting actin polymerization (Lawson and Ridley 2018; SenGupta, Parent, and Bear 2021); RhoA is accumulated at the front probably via Cdc42 (Figure 5h,i) and also via Rac1 (Nanda et al. 2023); and the Rho‐activated kinase ROCK is recruited to the leading edge by RhoA and the Cdc42‐binding protein aPKC, which may drive actomyosin contractility at the pseudopod and also promote correct pseudopod orientation for directionality control during chemotaxis.

## 4 Experimental Procedures

### 4.1 Antibodies

The anti‐Cdc42 (44; #610929) and anti‐PKCι/λ (41; #610207) mouse monoclonal antibodies were purchased from BD Transduction Laboratory; the anti‐RhoA (26C4; #sc‐418), anti‐GAPDH (0411; #sc‐47724), and anti‐β‐actin (C4; #sc‐47778) mouse monoclonal antibodies from Santa Cruz Biotechnology; the anti‐RhoA mouse monoclonal antibody (1B12; #ab54835), the anti‐ROCK1 rabbit monoclonal antibody (EPR638Y; #ab134181), and anti‐ROCK2 rabbit polyclonal antibody (#ab71598) from Abcam; the anti‐FLAG mouse monoclonal antibody (M2; #F1804) and anti‐FLAG rabbit polyclonal antibody (#F7425) from Sigma‐Aldrich; the anti‐Myc mouse monoclonal antibody (9E10; #11667203001) from Roche Applied Science; and the anti‐phospho‐Ser‐PKC substrate (#2261) and anti‐phospho‐MLC2 (pMLC) (Thr18/Ser19; #3674) rabbit polyclonal antibodies, the anti‐pMLC2 mouse monoclonal antibody (Ser19; #3675), and the anti‐phospho‐ezrin (Thr567)/radixin (Thr564)/moesin (Thr558) (pERM) rabbit monoclonal antibody (48G2; #3726) from Cell Signaling Technology. The anti‐PKCζ rabbit polyclonal antibody (#sc‐216) obtained from Santa Cruz Biotechnology is referred to “anti‐pan‐aPKC antibody” in the present study because this antibody recognizes both aPKCζ and aPKCι, the two isoforms of aPKC (Tocan et al. 2021).

### 4.2 Plasmids

The cDNAs encoding the following human proteins were prepared as previously described: aPKCι (amino acids 1–587) (Noda et al. 2003) and Par6β (amino acids 1–372) (Noda et al. 2001), and ROCK1 (amino acids 1–1354) (Ishizaki et al. 1996; Takeya et al. 2008). The cDNA for human MLC2 (amino acids 1–172) was prepared by PCR using Human Multiple Tissue cDNA panels (Takara Bio Inc.). The constitutively active form of aPKC (aPKCι‐ΔPS) that lacks the pseudosubstrate region (amino acids 106–127) was prepared by PCR using the cDNA for full‐length aPKCι as a template. The cDNA fragments for various regions of aPKCι and ROCK1 were amplified by PCR using specific primers. Mutations leading to the indicated amino acid substitutions or deletions were introduced by PCR‐mediated site‐directed mutagenesis. The cDNAs were ligated to the following expression vectors: pEF‐BOS (Mizushima and Nagata 1990) or pcDNA3.1(+) (Thermo Fisher Scientific) for expression in mammalian cells; and pGEX‐6P (GE Healthcare Biosciences) for expression as GST‐fusion protein in *Escherichia coli*. All of the constructs were sequenced for confirmation of their identities.

### 4.3 Reagents

C5a was purchased from R&D Systems, fMLP from Sigma‐Aldrich, Y‐27632 from Millipore, and human fibronectin from FUJIFILM Wako Pure Chemical Corporation.

### 4.4 Cell Culture and Differentiation of PLB‐985 Cells

In the present study, neutrophil‐differentiated PLB‐985 cells but not undifferentiated PLB‐985 cells were used in all of the analysis using chemoattractants. PLB‐985 cells were grown in RPMI 1640 (Nissui) supplemented with 25 mM HEPES (Nacalai Tesque), 10% FBS. For differentiation toward the neutrophil lineage, PLB‐985 cells were plated at a density of 2.0 × 10^5^ cells/mL in the fresh medium with 1.25% DMSO (Nacalai Tesque) and cultured for 6 days.

Human embryonic kidney HEK293T cells were cultured in Dulbecco's modified Eagle's medium (Nissui) supplemented with 10% FBS and penicillin/streptomycin.

### 4.5 Knockdown With siRNA

Double‐stranded, small interfering RNAs (siRNA) targeting human Cdc42, aPKC, RhoA, ROCK1, and ROCK2 containing the following sequences on the sense strand of 25‐nucleotide modified synthetic RNAs (Stealth RNAi; Thermo Fisher Scientific) were used: Cdc42 siRNA‐1, 5′‐CCUCUACUAUUGAGAAACUUGCCAA‐3′ (sense) and 5′‐UUGGCAAGUUUCUCAAUAGUAGAGG‐3′ (antisense); Cdc42 siRNA‐2, 5′‐UCCUUUCUUGCUUGUUGGGACUCAA‐3′ (sense) and 5′‐UUGAGUCCCAACAAGCAAGAAAGGA‐3′ (antisense); aPKC siRNA‐1, 5′‐GGGUACAGACAGAGAAGCACGUGUU‐3′ (sense) and 5′‐AACACGUGCUUCUCUGUCUGUACCC‐3′ (antisense); aPKC siRNA‐2, 5′‐GAGCGAGGGAUAAUUUAUAGAGAUU‐3′ (sense) and 5′‐AAUCUCUAUAAAUUAUCCCUCGCUC‐3′ (antisense); RhoA siRNA‐1, 5′‐CAGGUAGAGUUGGCUUUGUGGGACA‐3′ (sense) and 5′‐UGUCCCACAAAGCCAACUCUACCUG‐3′ (antisense); RhoA siRNA‐2, 5′‐ACGUGCCCAUCAUCCUGGUUGGGAA‐3′ (sense) and 5′‐UUCCCAACCAGGAUGAUGGGCACGU‐3′ (antisense); ROCK1 siRNA‐1, 5′‐UCAGUCAGAAUUCACAGCUUGCUAA‐3′ (sense) and 5′‐UUAGCAAGCUGUGAAUUCUGACUGA‐3′ (antisense); ROCK1 siRNA‐2, 5′‐GCAUUUGGAGAAGUUCAAUUGGUAA‐3′ (sense) and 5′‐UUACCAAUUGAACUUCUCCAAAUGC‐3′ (antisense); ROCK2 siRNA‐1, 5′‐GAAGCAGCUAUUAACAGAAAGAACA‐3′ (sense) and 5′‐UGUUCUUUCUGUUAAUAGCUGCUUC‐3′ (antisense); ROCK2 siRNA‐2, 5′‐GGAGGAGAUUAUAGCACCUUGCAAA‐3′ (sense) and 5′‐UUUGCAAGGUGCUAUAAUCUCCUCC‐3′ (antisense). It should be noted that the nucleotide sequences of both aPKC siRNA‐1 and aPKC siRNA‐2 were derived from those shared by the two human aPKC isoforms aPKCι and aPKCζ. Stealth RNAi siRNA Negative Control Med GC Duplex #2 (Thermo Fisher Scientific) was used as a negative control.

For siRNA transfection, PLB‐985 cells were differentiated for 3 days with the RPMI medium containing 1.25% DMSO and then nucleofection was performed using Human Monocyte Nucleofector Kit (Lonza). 1.0 × 10^7^ cells resuspended in 100 μL of the monocyte nucleofector solution were mixed with 30 ng of siRNA, and the cell‐siRNA mixture was subjected to nucleofection (program Y‐01) using the Nucleofector apparatus (Lonza). After incubation for 2 h in the LGM‐3 medium (Lonza), the medium was replaced with the RPMI medium containing 1.25% DMSO, and cells were cultured for another 3 days before use in further analysis.

### 4.6 Immunofluorescence Microscopy

Immunofluorescence microscopy was performed as previously described (Hayase et al. 2013; Kamakura et al. 2013, 2024; Horikawa et al. 2024). For preparation of FLAG–aPKCι‐expressing PLB‐985 cells, transfection with its encoding plasmid was performed using the Human Monocyte Nucleofector Kit (Lonza). Differentiated PLB‐985 cells (1.0 × 10^7^) resuspended in 100 μL of the monocyte nucleofector solution were mixed with 5 μg of DNA. The cell‐DNA mixture was subjected to nucleofection (program Y‐01) using the Nucleofector apparatus (Lonza), and cells were incubated for 2 h in LGM‐3 medium (Lonza) before use in further experiments.

For staining of RhoA and Cdc42 with the anti‐RhoA (26C4 or 1B12) antibody and anti‐Cdc42 (44) antibody, respectively, differentiated PLB‐985 cells were fixed for 10 min in ice‐cold 10% TCA. Of note, our careful examinations revealed that other fixatives, including formaldehyde and methanol, were not suitable for visualization of RhoA and Cdc42 in differentiated PLB‐985 cells with the above‐mentioned specific antibodies. For staining of ROCK1, pMLC2, and FLAG‐tagged proteins, cells were fixed for 10 min in 3.7% formaldehyde. Fixed cells were then permeabilized and blocked with 0.1% Triton X‐100 in PBS (137 mM NaCl, 2.7 mM KCl, 8.1 mM Na_2_HPO_4_, and 1.5 mM KH_2_PO_4_, pH 7.4) containing 3% BSA for 20 min. Indirect immunofluorescence analysis was performed using the following secondary antibodies: Alexa Fluor 488‐labeled anti‐rabbit or anti‐mouse IgG antibodies; Alexa Fluor 594‐labeled anti‐rabbit or anti‐mouse IgG antibodies (Thermo Fisher Scientific). Actin filaments were stained with Alexa Fluor 594 Phalloidin (Thermo Fisher Scientific), and nuclei were stained with Hoechst 33342 (Thermo Fisher Scientific).

Confocal images were captured at room temperature with the confocal microscopes LSM700 (Carl Zeiss), LSM780 (Carl Zeiss), or A1R HD25 (Nikon), followed by analysis with ZEN (Carl Zeiss) or NIS elements (Nikon). The microscope LSM700 was equipped with a Plan‐Apochromat 20×/0.8 numerical aperture (NA) dry objective lens and a Plan‐Apochromat 63×/1.4 NA oil‐immersion objective lens. The microscope LSM780 was equipped with a Plan‐Apochromat 40×/1.3 NA oil‐immersion and a C‐Apochromat 63×/1.2 NA water‐immersion objective lens. The microscope A1R HD25 was equipped with a Plan‐Apochromat 40×/1.25 NA silicon‐immersion objective lens and a Plan‐Apochromat 60×/1.27 NA water‐immersion objective lens.

### 4.7 Super‐Resolution Imaging and Analysis

Super‐resolution microscopic analysis was performed as previously described (Tocan et al. 2021). SIM images were taken with an N‐SIM system (Nikon) attached to a Ti2‐E inverted microscope (Nikon) with a CMOS camera (ORCA‐Flash 4.0 V3; Hamamatsu Photonics) using a Plan Apochromat 100×/1.35 NA silicon‐immersion objective lens at a step size of 0.12 μm. The chromatic aberration of the system was calibrated and corrected by 0.1 μm TetraSpeck beads (T7279; Thermo Fisher Scientific) in the mounting medium. Images were reconstructed and analyzed using NIS elements AR (Nikon) according to the manufacturer's protocol.

### 4.8 Transient Cell Polarization Induced With a Uniformly‐Applied Chemoattractant

For analysis of transiently‐polarized cells by uniform stimulation with C5a or fMLP, differentiated PLB‐985 cells suspended in a modified Hanks' buffer (140 mM NaCl, 5.4 mM KCl, 1 mM Tris–HCl, 1.1 mM CaCl_2_, 0.4 mM MgSO_4_, 1 mM HEPES, pH 7.2) containing 0.5% BSA were allowed to adhere to glass bottom dishes for 10 min at 37°C. After the preincubation, C5a (3 nM) or fMLP (100 nM) was uniformly applied to cells on the dish and incubated for 90 s at 37°C. Cells were fixed according to the above‐described protocol for analysis by immunofluorescence microscopy.

### 4.9 Zigmond Chamber Chemotaxis Assay

For analysis of persistently polarized cells in a gradient of C5a, the Zigmond chamber chemotaxis assay was performed. Differentiated PLB‐985 cells suspended in a modified Hanks' buffer containing 0.5% BSA were allowed to adhere to glass coverslips for 10 min at 37°C. The coverslips were rinsed and placed on a Zigmond chamber (Neuro Probe) (Zigmond 1977). Aliquots of the modified Hanks' buffer were added to one side of the chamber, whereas those of 3 nM C5a solution in the same buffer were added to the other side. The chambers were placed for 10 min at 37°C on a heated stage. Cells were then fixed for immunostaining. Pseudopod orientation toward C5a was analyzed by actin staining: cells in which actin accumulates within a 120° arc facing the source of C5a are regarded as ones with correct pseudopod orientation (mean ± SD of three independent experiments; 100–300 of polarized cells were analyzed in each experiment). “Polarized cells” were defined as those with a single actin‐rich protrusion (a pseudopod).

### 4.10 Transwell Chemotaxis Assay

Transwell chemotaxis assays were performed using 24‐well transwell chambers (pore size, 3.0 μm; Corning) as previously described (Kamakura et al. 2013). After incubation for 45 min at 37°C, the migrated cells into the lower chamber were counted using a hemocytometer, and the percentage of migrating neutrophils was calculated by dividing the number of cells in the lower chamber by that of the input cells.

### 4.11 Protein Identification by LC–MS/MS Analysis

For identifying novel aPKC‐interacting proteins, HEK293T cells were transfected with a plasmid vector encoding FLAG‐tagged aPKCι (K274E) or with an empty FLAG vector. Transfected cells were cultured for 48 h and then lysed at 4°C with a lysis buffer (150 mM NaCl, 5 mM EDTA, 1 mM DTT, 0.5% Triton X‐100, 10% glycerol, and 50 mM Tris–HCl, pH 7.5) containing Protease Inhibitor Cocktail (Sigma‐Aldrich). Proteins in the lysates were precipitated with the anti‐FLAG (M2) antibody‐conjugated magnetic beads (Sigma‐Aldrich). After washing three times with the lysis buffer, the proteins were subjected to SDS‐PAGE, followed by silver staining. Protein identification using LC–MS/MS was performed at the Laboratory for Research Support, Medical Institute of Bioregulation, Kyushu University, according to the protocol of Matsumoto et al. (2017). Bands separated by SDS‐PAGE were excised from the gel, and the proteins in the gel were digested with Trypsin gold Mass Spectrometry grade (PROMEGA) dissolved in 25 mM ammonium bicarbonate solution. The gel‐extracted peptides were subjected to nano‐LC–MS/MS analysis using a Finnigan LTQ mass spectrometer (Thermo Fisher Scientific). MS/MS spectra were obtained automatically in a data‐dependent scan mode and compared with those in the UniProtKB/SwissProt human peptide database (UniProt Consortium) using the MASCOT search engine (Matrix Science). Assigned high‐scoring peptide sequences were manually confirmed by comparison with the corresponding spectra.

### 4.12 Immunoprecipitation Analysis

Immunoprecipitation was performed as previously described (Kamakura et al. 2024; Kohda et al. 2024). Briefly, HEK293T cells were transfected with the indicated cDNAs using X‐tremeGENE HP DNA Transfection Reagent (Roche), followed by culture for 48 h. Cells were then lysed with a lysis buffer (150 mM NaCl, 5 mM EDTA, 1 mM DTT, 0.1% Triton X‐100, 10% glycerol, and 50 mM Tris–HCl, pH 7.5) containing Protease Inhibitor Cocktail (Sigma‐Aldrich) for analysis of the interaction of aPKC (KE) and ROCK1‐C; or with another lysis buffer (150 mM NaCl, 5 mM EGTA, 2 mM MgCl_2_, 10 mM β‐glycerophosphate, 5 mM NaF, 1 mM NaPPi, 1 mM Na_3_VO_5_, 1 mM DTT, 0.1% Triton X‐100, 10% glycerol, and 50 mM Tris–HCl, pH 7.5) containing Protease Inhibitor Cocktail for analysis of phosphorylation of ROCK1. Proteins in the lysates were precipitated with the anti‐FLAG (M2) antibody, anti‐Myc (9E10) antibody, or control IgG1, coupled to protein G‐Sepharose (GE Healthcare Biosciences). The precipitants were analyzed by immunoblot with the indicated antibodies, and the blots were developed using ImmunoStar Zeta or ImmunoStar LD (FUJIFILM Wako) for visualization.

### 4.13 In Vitro Kinase Assay

GST‐tagged MLC2 protein was expressed in *E. coli* BL21 (Agilent Technologies) and purified with glutathione–Sepharose–4B (GE Healthcare). HEK293T cells were transfected with a plasmid encoding FLAG–ROCK1 or FLAG–aPKCι (wt or K274E), and cultured for 24 h. Cells were lysed at 4°C with a lysis buffer (150 mM NaCl, 5 mM EGTA, 2 mM MgCl_2_, 10 mM β‐glycerophosphate, 5 mM NaF, 1 mM NaPPi, 1 mM Na_3_VO_5_, 1 mM DTT, 0.1% Triton X‐100, 10% glycerol, and 50 mM Tris–HCl, pH 7.5 at 4°C) containing Protease Inhibitor Cocktail (Sigma‐Aldrich). The FLAG‐tagged protein in the lysate was precipitated with the anti‐FLAG M2 antibody coupled to protein G‐Sepharose beads (GE Healthcare), and then washed twice with the lysis buffer and once with a kinase reaction buffer (10 mM MgCl_2_, 2 mM EGTA, 10% glycerol, 2 mM DTT, 20 mM Tris–HCl, pH 7.5 at 25°C). For kinase assay, the FLAG–aPKCι‐bound beads were mixed with the FLAG–ROCK1‐bound beads in the kinase reaction buffer containing 2.8 μM GST–MLC2 and 100 μM ATP, followed by incubation at 30°C for 10 min. The kinase reaction was stopped by the addition of Laemmli's sample buffer. Phosphorylation of GST–MLC2 was detected by immunoblot analysis with the anti‐pMLC2 antibody.

### 4.14 Statistical Analysis

Statistical differences were analyzed by one‐way ANOVA with Tukey–Kramer's multiple comparisons of the means test.

## Author Contributions

**Atsushi Naito:** conceptualization, investigation, methodology, visualization, writing – review and editing, writing – original draft, data curation, formal analysis. **Sachiko Kamakura:** conceptualization, data curation, formal analysis, visualization, writing – original draft, writing – review and editing, supervision, investigation, methodology, funding acquisition, resources, project administration. **Junya Hayase:** data curation, formal analysis, visualization, writing – review and editing, methodology, investigation. **Akira Kohda:** data curation, formal analysis, resources, supervision, writing – review and editing. **Hiroaki Niiro:** supervision, writing – review and editing. **Koichi Akashi:** supervision, writing – review and editing. **Hideki Sumimoto:** conceptualization, data curation, formal analysis, writing – original draft, writing – review and editing, project administration, supervision, methodology, funding acquisition, resources.

## Conflicts of Interest

The authors declare no conflicts of interest.

## Data Availability

The data that support the findings of this study are available on request from the corresponding author. The data are not publicly available due to privacy or ethical restrictions.
